# Patient-Reported Anxiety/Depression After Endovascular Thrombectomy: A *post-hoc* Analysis of Direct-MT Trial

**DOI:** 10.3389/fneur.2022.811629

**Published:** 2022-02-09

**Authors:** Ping Zhang, Hong-jian Shen, Lei Chen, Xuan Zhu, Min-min Zhang, Yi Jiang, Peng-fei Yang, Lei Zhang, Peng-fei Xing, Xiao-fei Ye, Min Lou, Cong-guo Yin, Ben-qiang Deng, Tao Wu, Yong-wei Zhang, Jian-min Liu

**Affiliations:** ^1^Department of Neurovascular Center, Changhai Hospital, Naval Medical University, Shanghai, China; ^2^Department of Statistics, Naval Medical University, Shanghai, China; ^3^Department of Neurology, Second Affiliated Hospital of Zhejiang University, Hangzhou, China; ^4^Department of Neurology, Hangzhou First People's Hospital, Hangzhou, China

**Keywords:** anxiety, depression, ischemic stroke, thrombolysis, thrombectomy

## Abstract

**Objective:**

To analyze the effect of endovascular thrombectomy (EVT) alone vs. EVT after an intravenous (IV) alteplase of ischemic stroke on a patient-reported anxiety/depression, and to identify predictors of patient-reported anxiety/depression by analyzing data from Direct Intraarterial Thrombectomy in Order to Revascularize the patients with Acute Ischemic Stroke with a Large Vessel Occlusion Efficiently in Chinese Tertiary Hospitals: a Multicenter Randomized Clinical Trial (DIRECT-MT).

**Methods:**

Patients with acute ischemic stroke (AIS), triggered by a large-vessel occlusion in the anterior circulation, were randomly allocated to undergo an EVT after IV alteplase (combination-therapy group) or an EVT alone (EVT-alone group) at a 1:1 ratio in DIRECT-MT. Patients in both groups were followed up for 90 days (±14 days) after stroke using a structured modified Ranking Scale (mRS), a Barthel Index (BI), and a 5-Dimensional European Quality of Life Scale (EQ-5D-5L). Patients who returned EQ-5D-5L were included. The EQ-5D-5L anxiety/depression dimension was used to analyze the patient-reported anxiety/depression. First, differences in patient-reported anxiety/depression were compared between the combination-therapy group and the EVT-alone group. Then, the baseline and influencing factors between the anxiety/depression group and no anxiety/depression group were analyzed using univariate regression analysis. Finally, variables with *p* < 0.1 in univariate regression were subjected to multivariable binary regression analysis to screen independent predictors for patient-reported anxiety /depression after ischemic stroke.

**Results:**

**:** Five hundred fifteen patients returned the EQ-5D-5L in Direct-MT. Of these patients, 226 (43.88%) reported a level of anxiety/depression, and about 7% reported a severe or extremely severe anxiety/depression. The patient-reported anxiety/depression in the EVT-alone group was significantly higher than that in the combination-therapy group (48.26% vs. 39.45%, *p* = 0.04). The clinical outcomes were significantly different between the no Anxiety/Depression Group and the anxiety/depression group (mRS at 90 days:2 *vs* 3, *p* < 0.001; BI of 95 or 100 at 90 days: 73.36% vs 42.04%, *p* < 0.001; EQ-5D-5l utility indexes at 90 days:0.96 vs.57, *p* < 0.001). Logistic regression analysis showed that allocation to thrombolysis before EVT strategy was inversely associated with anxiety/depression [0.61(0.40, 0.94), *p* = 0.03], an insular cortex ischemia, and National Institute of Health Strocke Scale (NIHSS) at 7 days were positively associated with anxiety/depression [2.04(1.07, 3.90), *p* = 0.03; 1.07(1.03, 1.12), *p* < 0.001].

**Conclusions:**

Patient-reported anxiety/depression may suggest that there is a benefit to administering intravenous alteplase before EVT. It may also indicate that it is better to provide IV alteplase before EVT, rather than EVT alone according to patient-reported anxiety/depression. Future research should consider not only the motor function impairments but also the patient-reported mental problems as measures of treatment efficacy in patients with stroke (DIRECT-MT ClinicalTrials.gov number, NCT03469206).

## Introduction

Endovascular thrombectomy (EVT) has been demonstrated to be effective in patients with stroke with large vessel occlusion. However, there is uncertainty regarding the risk and benefit of administering intravenous alteplase before endovascular thrombectomy. The Direct-MT Trial showed that EVT alone was not inferior to EVT after the intravenous (IV) alteplase with regard to the modified Rankin Scale (mRS) score at 90 days (adjusted common odds ratio, 1.07; 95% confidence interval, 0.81–1.40; *p* = 0.04 for non-inferiority) (1). However, the mRS is largely biased toward motor function impairments and does not reflect other important domains of the health of patients, such as anxiety and depression. Although they have a mild disability represented in the mRS, patients with post-stroke may have mood disorders that significantly affect quality of life. Anxiety and depression are common after a stroke, occurring in about a third and a quarter of stroke survivors, respectively (2). Anxiety and depression are associated with a significantly increased risk of mortality in stroke survivors and reduction in their quality of life (3). Therefore, identifying factors affecting the anxiety/ depression after a stroke may provide valuable clues to a better understanding of treatment benefit.

The 5-Dimensional European Quality of Life Scale (EQ-5D-5L) was already applied and validated as a patient-reported outcome measure (PROMs) in patients with stroke (4). The PROMs was recognized by the European Medicines Agency (EMA) and the Food and Drug Administration (FDA) as a measure of treatment efficacy (5). The EQ-5D-5L anxiety/depression dimension was used to analyze the patient-reported feeling of anxiety/depression in our study. This study aims to evaluate whether there is any difference in the patient-reported anxiety/depression between a combination-therapy group and an EVT-alone in Direct-MT Trial, with the hope to identify factors affecting the patient-reported anxiety/ depression after a stroke.

## Patients and Methods

### Design and Participants

This study was a *post-hoc* analysis of DIRECT-MT Trial, including patients who were admitted to 41 academic tertiary care centers in 18 provinces of China between February 2018 and September 2019. All the included patients had ischemic stroke triggered by a large-vessel occlusion in the anterior circulation within 4.5 h after the symptom onset. All of them had neurological deficits with at least 2 points on the National Institutes of Health Stroke Scale (NIHSS). The baseline extent of cerebral ischemia was first measured by CT and then by the Alberta Stroke Program Early Computed Tomography Score (ASPECTS). Patients who met the inclusion criteria and exclusion criteria of DIRECT-MT Trial (2) were randomly allocated to a combination-therapy (EVT after IV alteplase) group or an EVT-alone group. Patients in both groups were followed up for 90 days (±14 days) after the stroke using a structured mRS, a Barthel Index (BI), and an EQ-5D-5L. This study only included the patients who returned the EQ-5D-5L form in DIRECT-MT Trial.

### Procedure

The clinical and demographic characteristics of the patients were obtained from their medical records. All outcome assessments were performed by locally trained neurologists who were blinded to the grouping. Patients were interviewed by telephone or in person with the use of standardized forms. Two members of the outcome committee verified the score by consensus with the standardized written reports of each interview.

In EQ-5D-5L, health is defined in five dimensions: Anxiety/Depression, Mobility, Self-Care, Usual Activities, and Pain/Discomfort. Each dimension has five levels: no problem (level 1), minor problems (level 2), moderate problems (level 3), severe problems (level 4), and extremely severe problems (level 5). We used the anxiety/depression dimension to analyze the factors associated with patient-reported anxiety/depression. The patients were divided into two groups: anxiety/depression group (including level 2, 3, 4, and 5) and no Anxiety/Depression Group (including level 1 only).

Written informed consent was obtained from all participants. The study was approved by the institutional review board and local ethical committee of the 41 academic tertiary care centers concerned.

### Statistical Analysis

All statistical analyses were performed using a Statistical Analysis System (SAS) software, version 9.2 (SAS Institute). The incidence of patient-reported feeling of anxiety/depression was compared between the combination-therapy group and the EVT-alone group. Then, the baseline and influencing factors between anxiety/depression group and no Anxiety/Depression Group were compared using univariate regression analysis. Finally, variables with *p* < 0.1 in univariate regression were subjected to multivariable binary regression analysis. Categorical variables are expressed as counts (%), continuous variables with normal distribution are expressed as means (SE), and continuous variables not normally distributed are expressed as median (IQR) values. Differences between the groups were calculated using the χ^2^ test, the *t*-test, or the Mann–Whitney *U* test as appropriate. Multivariable analysis for anxiety/depression was made using the enter method. Values of *p* < 0.05 were considered statistically significant.

## Results

**Figure 1:**
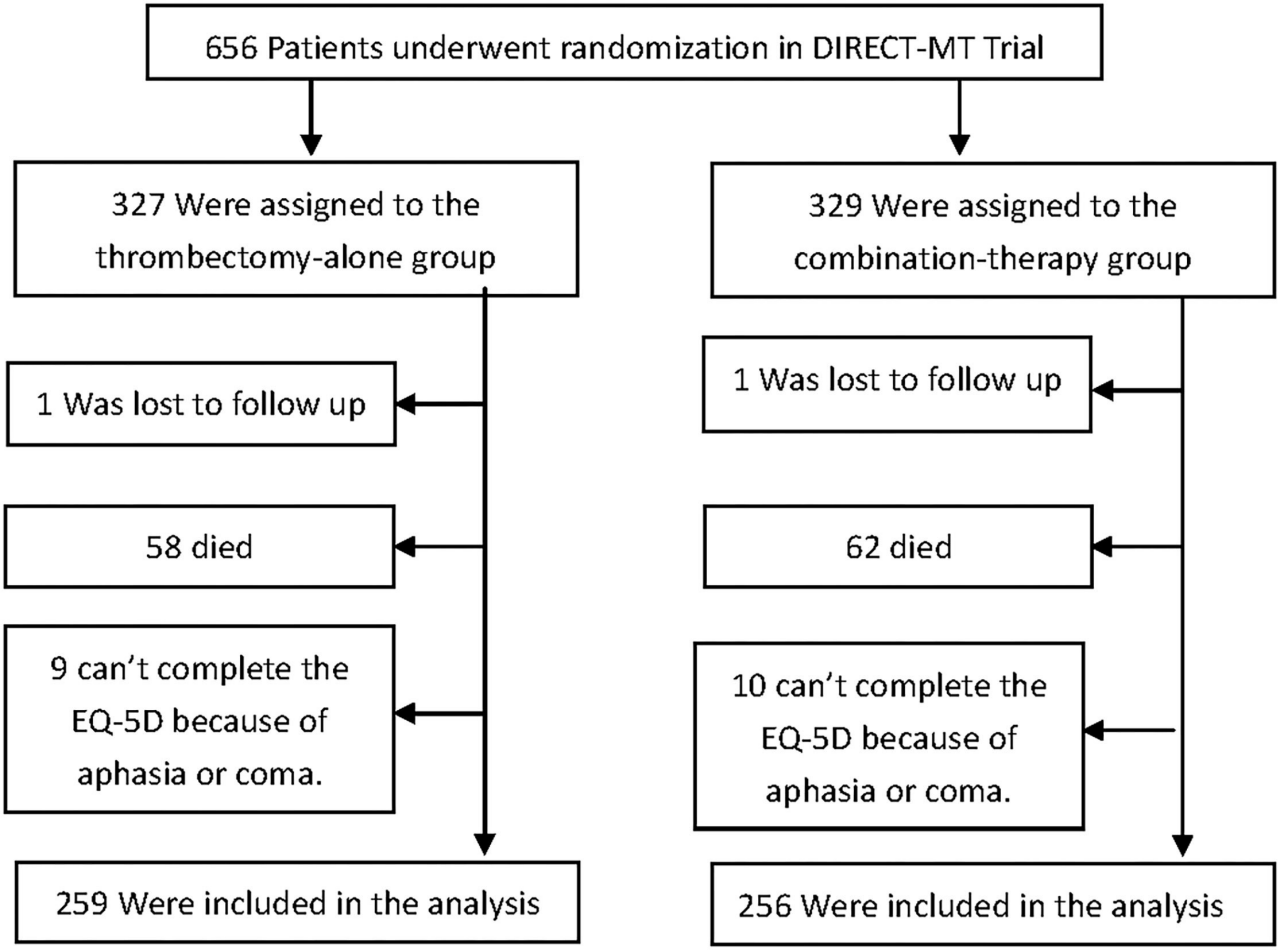
Randomization and treatment of the patients. 656 patients were randomly assigned in a 1:1 ratio to undergo endovascular thrombectomy (thrombectomy-alone group) or to receive intravenous (IV) alteplase followed by endovascular thrombectomy (combination-therapy group) in Direct-MT trial. Excluding the patients who lost follow up, died, aphasia and coma, a total of 515 patients were included in the analysis.

Of the 656 patients enrolled in Direct-MT Trial, 515 (78.51%) returned the EQ-5D-5L form (259 in EVT-alone group and 256 in combination-therapy group) (Figure 1). The patients who did not return the questionnaire had more severe strokes according to NIHSS upon hospital arrival [20 (3,39) vs. 16 (2,40), z=6.28, *p* < 0.001], and worse outcomes in mRS [6 (0,6) vs. 3 (0,5), z=17.61, *p* < 0.001] than those who returned the questionnaire.

**Figure 2:**
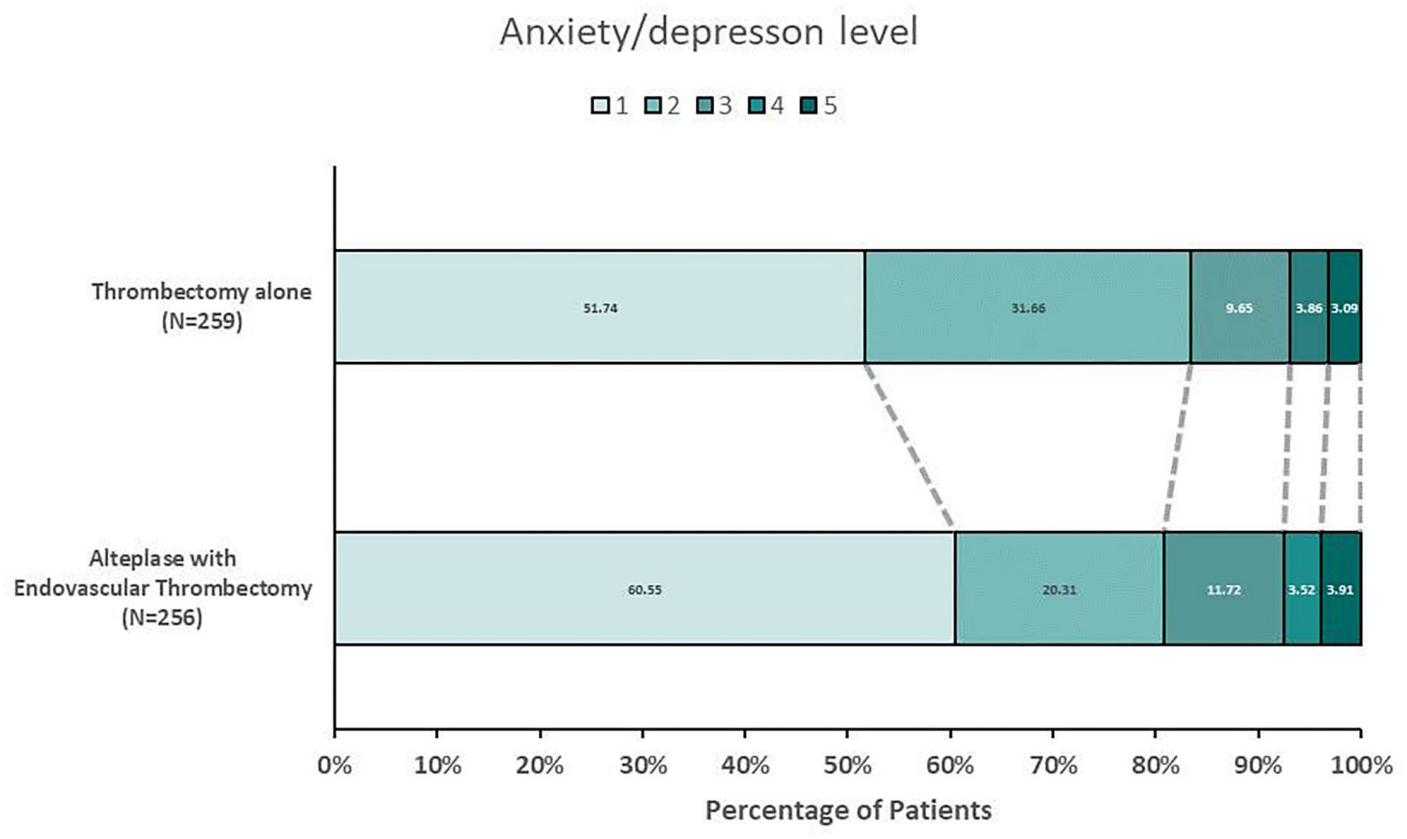
Distribution of patient-reported anxiety/depression in EQ-5D-5L in the Intention-to Treat Population. Shown are levels on the patient-reported anxiety/depression for the patients in the two treatment groups who returned EQ-5D-5L. Levels range from 1 to 5, with 1 indicating no anxiety/depression, 2 minor anxiety/depression, 3 moderate anxiety/depression, 4 severe anxiety/depression, and 5 extremely severe anxiety/depression. The incidence of patient-reported anxiety/depression (level 2,3,4,5) in Thrombectomy alone group was significantly higher than that in combination-therapy group (48.26% vs. 39.45%, P = 0.04).

Differences in patient-reported anxiety/depression between the EVT-alone group and combination-therapy group are shown in Figure 2. There were 226 patients (43.88%) who reported any level of anxiety/depression, and over 7% patients who reported severe or extremely severe depression. The incidence of patient-reported anxiety/depression in the EVT-alone group was significantly higher than in the combination-therapy group (48.26% vs. 39.45%, *p* = 0.04). The incidence of severe and extremely severe anxiety/depression was similar between the two groups (6.95% vs. 7.42%, *p* = 0.87).

The baseline characteristics of the Anxiety/Depression Group and the no Anxiety/Depression Group were analyzed (Table 1). It was found that the median score of NIHSS upon hospital arrival and NIHSS at 7 days in the Anxiety/Depression Group was higher than in the no Anxiety/Depression Group [15 (12,20) vs. 17 (13,21), *p* < 0.001; 3 (0,8) vs. 8 (3,14), *p* < 0.001)]. The number of patients treated with IV alteplase in the no Anxiety/Depression Group was 156 (53.98%) vs. 100 (44.25%) in the Anxiety/Depression Group (*p* = 0.03).

**Table 1:** Comparison of the baseline characteristics of patients between anxiety/depression group and no anxiety/depression group.

	**No anxiety/depression**	**Anxiety/depression**	***P***
No. of Patients	289	226	
Age, y; mean(SD)	66.87(11.50)	67.45(12.70)	0.36
male	177(61.25)	117(51.77)	0.03
smoking	74(25.61)	51(22.57)	0.42
Hypertension	162(56.06)	141(62.39)	0.15
Diabetes Mellitus	36(12.46)	44(19.47)	0.03
Atrial fibrillation	112(38.75)	110(48.67)	0.02
Hypercholesterolemia	13(4.50)	9(3.98)	0.77
Pre-stroke anxiety/depression	8(2.77)	6(2.65)	0.94
pre-stroke mRS	0(0,2)	0(0,2)	0.33
Glucose level at hospital arrival	7.15(2.13)	7.89(3.17)	0.02
Interval from stroke onset to revascular	265.50(209.50,315.00)	276.00(230.00,325.00)	0.07
NIHSS at hospital arrival	15(12,20)	17(13,21)	0.00
NIHSS at 7 days	3(0,8)	8(3,14)	0.00
**Cause of stroke**			
*Cardioembolic	109(37.72)	108(47.79)	0.10
*Intracranialatherosclerosis	21(7.27)	11(4.87)	
*Ipsilateralextracranial	35(12.11)	19(8.41)	
*Undetermined	124(42.91)	88(38.94)	
allocation to thrombolysis before EVT strategy	156(53.98)	100(44.25)	0.03

The possible imaging impact factors of anxiety/depression were analyzed (Table 2). The result showed that the cerebral infarction volume on CT scan in the Anxiety/Depression Group was larger than that of the no Anxiety/Depression Group [45.81 (14.90,110.85) vs. 25.44 (4.62,83.35), *p* < 0.001]. The location of cerebral ischemia was also different between the two groups. According to the baseline ASPECTS scores, the ischemic areas of the brain were divided into four parts: the basal ganglia, frontal lobe, parietal lobe, and insular cortex. The ischemia of the frontal lobe, parietal lobe, and insular cortex was more common in the Anxiety/Depression Group. The percentage of patients with successful reperfusion [expanded treatment in cerebral ischemia (eTICI) score, ≥2b] between the two groups were similar [181 (84.58) vs. 236 (86.45), *p* = 0.56]. The percentage of patients with asymptomatic intracranial hemorrhage in the Anxiety/Depression Group was higher than that of the no Anxiety/Depression Group [92 (40.71) vs. 76 (26.30), *p* < 0.001].

**Table 2:** Imaging characteristics of patients in anxiety/depression group and no anxiety/depression group.

	**No anxiety/depression**	**Anxiety/depression**	***P***
Baseline ASPECTS	9.00(7.00,10.00)	9.00(7.00,10.00)	0.16
**Ischemic site according to ASPECTS**			
*Basal ganglia	203(78.68)	179(85.24)	0.07
*Frontal lobe	92(35.66)	95(45.24)	0.04
*Parietal lobe	150(58.14)	149(70.95)	0.00
*Insular cortex	147(56.98)	151(71.90)	0.00
**Side of target occlusion**			
* left	122(43.73)	109(49.55)	0.47
* right	153(54.84)	106(48.18)	
* left and right	2(0.72)	2(0.91)	
*NA	2(0.72)	3(1.36)	
Collateral score (0–1)	209(72.32)	170(75.22)	0.46
**Location of intracranial artery occlusion**			
*ICA	88(30.88)	70(32.11)	0.67
*M1	162(56.84)	123(56.42)	
*M2	35(12.28)	24(11.01)	
eTICI score assessed on final angiogram≥2b	236(86.45)	181(84.58)	0.56
Cerebral infarction volume on CT: Median(IQR)	25.44(4.62,83.35)	45.81(14.90,110.85)	0.00
Symptomatic Intracranial hemorrhage	5(1.73)	3(1.33)	0.99
asymptomatic intracranial hemorrhage	76(26.30)	92(40.71)	0.00

Multivariable regression analysis showed that allocation to thrombolysis before EVT strategy, ischemia of the insular cortex, and NIHSS at 7 days were independent predictors for anxiety/depression (Table 3). Allocation to thrombolysis before EVT strategy was inversely associated with anxiety/depression [0.61(0.40,0.94), *p* = 0.03]. Insular cortex ischemia and NIHSS at 7 days were positively associated with anxiety/depression [2.04(1.07, 3.90), *p* = 0.03; 1.07(1.03,1.12), *p* < 0.001].

**Table 3:** Multivariable binary logistic regression analysis of variables associated with anxiety/depression in EQ-5D-5L at 90 days (*n* = 515).

**Variable**	**OR(95%CI)**	***P*-value**
allocation to thrombolysis before EVT strategy	0.61(0.40,0.94)	0.03
Insular cortex ischemia	2.04(1.07,3.90)	0.03
NIHSS at 7 days	1.07(1.03,1.12)	0.00

The clinical outcomes of the Anxiety/Depression Group and the no Anxiety/Depression Group were significantly different (Table 4). The median score of mRS score at 90 days was 3 in the Anxiety/Depression Group vs. 2 in the no Anxiety/Depression Group (*p* < 0.001). Patients in the Anxiety/Depression Group were more dependent than those in no Anxiety/Depression Group (mRS score 3-5: 73.45% vs. 38.06%, *p* < 0.001). Patients with anxiety/depression had lower activities of daily living (ADL) [BI: 95 or 100 at 90 days-no (%), (Anxiety/Depression Group) 95 (42.04%) vs. (no Anxiety/Depression Group) 212(73.36%); *p* < 0.001]. The EQ-5D-5L utility index in patients with anxiety/depression was lower than that in patients with no anxiety/depression [0.57 (0.06,0.85) vs. 0.96 (0.78,1), *p* < 0.001]. Lastly, the EQ-5D-5L utility index was inversely associated with the mRS (ρ_s_=-0.87, *p* < 0.001).

**Table 4:** Clinical outcomes in patients of anxiety/depression group and no anxiety/depression group.

	**No anxiety/depression**	**Anxiety/depression**	***P***
mRS at 90 days-Median (IQR)	2(0,3)	3(2,5)	0.00
mRS 3-5 at 90 days-no(%)	110(38.06)	166(73.45)	0.00
Barthel Index of 95 or 100 at 90 days-no(%)	212(73.36)	95(42.04)	0.00
EQ-5D-5l utility indexes at 90 days-Median(IQR)	0.96(0.78,1.00)	0.57(0.06,0.85)	0.00

## Discussion

Patient-reported outcome measures (PROMs) have become an important tool to measure the quality of interventions. Compared with mRS, which is largely based on motor function, the EQ-5D-5L represents important health domains like Anxiety/Depression. According to mRS, the results do not rule out the benefit of alteplase in DIRECT-MT (2). When we assessed the patient-reported anxiety/depression, essential information on the outcome in patients with stroke after EVT was present. It is a valuable outcome parameter after an EVT complementary to the mRS (6).

Of the 515 patients, 226 (43.88%) reported some level of anxiety/depression, and about 7% patients reported severe or extremely severe anxiety/depression. The incidence of anxiety/depression observed in our study is similar to what was reported in a meta-analysis from King's College London (7), in which anxiety was present in 9.8% of patients with stroke and depressive disorder was present in 33.5% of patients with stroke, with an overall anxiety/depression incidence of 43.3%. Sallinen et al. found that 44% patients had depression 3 months after intracerebral hemorrhage (8). As different studies used different scales and cohorts, the reported incidences of anxiety/depression in patients with stroke are not all the same. However, all of them pointed out that the incidence of post-stroke anxiety/depression was very high and should be taken seriously.

It is noteworthy that the incidence of anxiety/depression in the EVT-alone group of our study was significantly higher than in the combination-therapy group (48.26% vs. 39.45%, *p* = 0.04). Multivariable regression analysis showed that allocation to thrombolysis before MT strategy was inversely associated with anxiety/depression, indicating that IV thrombolysis can reduce the incidence of anxiety/depression after cerebral infraction. The effect of IV thrombolysis on reducing anxiety/depression was also observed in the Wake-up trial. It was found (9) that with and without adjustment for age and stroke severity, intravenous alteplase resulted in a significant reduction of the proportion of patients on post-stroke depression 90 days after stroke. Hence, reducing the incidence of anxiety/depression is more likely to be the biological effects of alteplase. We hypothesize that there are several reasons for the result. First, IV alteplase can act in small vessels before and after thrombectomy for the reperfusion of these small vessels. This may be the reason why we observed a higher percentage of patients with successful reperfusion in the combination-therapy group as compared with that in the EVT-alone group before thrombectomy (2). Reperfusion also leads to salvaging brain tissue from final infarction. The incidence of the main side effect of alteplase, symptomatic hemorrhage, showed no difference between the anxiety/depression group and the no anxiety/depression group. All of these may be responsible for the lower incidence of anxiety/depression in the combination-therapy group. Secondly, the fibrinolytic system is considered as a new target for depression (10). Tissue plasminogen activator (tPA) is considered as a key factor that does not only promote fibrinolysis but also regulates synaptic plasticity, neurogenesis, and neurite outgrowth, especially through the tissue plasminogen activator–brain-derived neurotrophic factor (tPA-BDNF) pathway (11, 12). A genetic research found that genetic variations in the p11/tPA/BDNF pathway play a central role with post-stroke depression (13). Clinical research found that serum tPA was decreased in depressive patients, and the effect was reversed with antidepressant treatment, suggesting that it is associated with depression pathogenesis (12). Therefore, alteplase administration before thrombectomy may be a better way than thrombectomy alone to reduce the incidence of anxiety/depression after stroke. Thirdly, anxiety/depression was not easily captured by the mRS score in Chinese patients. Due to the introverted nature of Chinese people, many patients were reluctant to admit that emotional disorders already affect their ability to live. However, EQ-5D-5L anxiety/depression dimension has five levels and are more likely to be accepted by patients (14). Symptoms of patients with stroke often cannot be captured by “classical” outcome measures. Therefore, measures of emotion should be included as an essential part in future studies.

In our trial, it was found that patients with self-reported anxiety/depression had poor outcomes, such as high mRS, lower BI, and lower EQ-5D-5L utility index. The MRS, BI, and EQ-5D-5L were all outcomes which were evaluated 90 days after a stroke. They are statistically related through association, rather than through causation. Worsened social function may cause anxiety/depression and an anxiety/depression may also cause a worse social function. Interestingly, the NIHSS at 7 days, not the NIHSS upon hospital arrival, was positively associated with anxiety/depression, which may be a benefit in intravenous alteplase. This result indicated that the degree of neurological impairment after treatment was an independent cause of anxiety/depression. Therefore, we should pay more attention to the patients with severe neurological deficits. More detailed information on post-stroke anxiety/depression is needed in further studies, which may influence social function and living qualities of patients.

This study has some limitations. First, patient-reported anxiety/depression was only evaluated by EQ-5D-5L, and it cannot distinguish between anxiety and depression. Although EQ-5D-5L has been shown as a valid measure of generic health outcomes, especially psychometric advantages after stroke (4, 14), as EQ-5D-5L is not an instrument to diagnose anxiety or depression. Second, we only included the patients who returned the EQ-5D-5L form and acute ischemic stroke (AIS) caused by large-vessel occlusion in the anterior circulation. Third, we lacked the analysis of socioeconomic factors which may affect the emotional state of a patient.

## Conclusions

The present study suggests that there is some benefits to administering intravenous alteplase before EVT. It may be better to provide IV alteplase before EVT rather than EVT alone among patients with AIS, within 4.5 h after the symptom onset with a large-vessel occlusion in the anterior circulation according to patient-reported anxiety/depression. Future research should consider not only the motor function impairments but also the patient-reported mental problems as measures of treatment outcomes in patients with stroke.

## Data Availability Statement

The original contributions presented in the study are included in the article/supplementary material, further inquiries can be directed to the corresponding author/s.

## Ethics Statement

The studies involving human participants were reviewed and approved by Shanghai Changhai Hosptial Ethics Committee. The patients/participants provided their written informed consent to participate in this study.

## Author Contributions

All authors listed have made a substantial, direct, and intellectual contribution to the work and approved it for publication.

## Funding

This work was funded by a grant (GN-2017R0001) from the Stroke Prevention Project of the National Health Commission of the Peoples Republic of China and by the Wu Jieping Medical Foundation. The publication fee was funded by Shanghai key clinical specialty (shslczdzk06101).

## Conflict of Interest

The authors declare that the research was conducted in the absence of any commercial or financial relationships that could be construed as a potential conflict of interest.

## Publisher's Note

All claims expressed in this article are solely those of the authors and do not necessarily represent those of their affiliated organizations, or those of the publisher, the editors and the reviewers. Any product that may be evaluated in this article, or claim that may be made by its manufacturer, is not guaranteed or endorsed by the publisher.
